# Does the Brain Prefer Geometrical Homogeneity?

**DOI:** 10.3233/BEN-2010-0263

**Published:** 2010-11-23

**Authors:** A. Midorikawa, M. Kawamura

**Affiliations:** ^1^Department of PsychologyChuo University Faculty of LettersTokyoJapan; ^2^Department of NeurologyShowa University School of MedicineTokyoJapan

**Keywords:** Dementia, frontotemporal lobar degeneration (FTLD), residual function, geometrical ability

## Abstract

Some patients with frontotemporal lobar degeneration (FTLD) have shown the development of painting or musical abilities after the onset of the disease. In this report, we present another emergent ability. A female patient with FTLD showing dense atrophy of the bilateral anterior lobes and a loss of voluntary activity in aspects of daily living, presented with the characteristic behaviours when given a paper and a pair of scissors. When a shape was already drawn on the paper, she showed reasonable skills with the scissors, cutting without any hesitation. When she cut a blank piece of paper, she showed quite unique geometrical preferences. Her severely degenerated brain combined with her geometrical abilities suggests that the human brain is naturally affected by geometrical homogeneity.

## 1. Introduction

Dementia deprives us of various abilities, not only of memory function, but also verbal, motor, cognitive, or executive functions [2] For a long time it had been postulated that a demented brain gradually robbed someone of his or her basic functions, leading to a person without abilities. Recently, however, several studies have shown that even after the onset of dementia some patients show a development or emergence of several different abilities, such as painting [5,7,14,15,18, 21], composing music, photography, or solving a puzzle [16,17]. Some famous musical compositions were actually a product of dementia [1]. Although there are several subtypes of dementia, all of the aforementioned patients were diagnosed with, or considered to have frontotemporal lobar degeneration (FTLD), which presents as primary dementia characterized by disproportionate atrophy of the anterior frontal and temporal lobes [8]. Accordingly, a deficit of the anterior portion of the brain is considered crucial for the preservation or emergence of several abilities. However, some of these reported cases had left-sided asymmetrical atrophy, suggesting that specific latent or novel abilities are restricted to left-sided asymmetrical atrophy.

In this article, we present a case of bilateral dense atrophy of the frontotemporal lobes coinciding with newly discovered latent or novel abilities. We believe that investigation of these abilities might be a gateway to hidden human functions.

## 2. Case history

In December 2002, a 66-year-old female patient was admitted to Showa University Hospital in Tokyo, Japan, because of severe word-finding difficulty and a substantial change in personality. Her symptoms had begun at the end of the year 2000, and her neurological examination (conducted at another hospital) was normal then, with the exception for verbal impairment. Her spontaneous speech had decreased, but she was capable of shopping and preparing a meal by herself. Over the next two years, her condition markedly deteriorated. She was unable to perform most spontaneous activities, but was still able to follow instructions, walk, and eat, independently. In February 2003,

ISSN 0953-4180/10/$27.50 © 2010 – IOS Press and the authors. All rights reserved

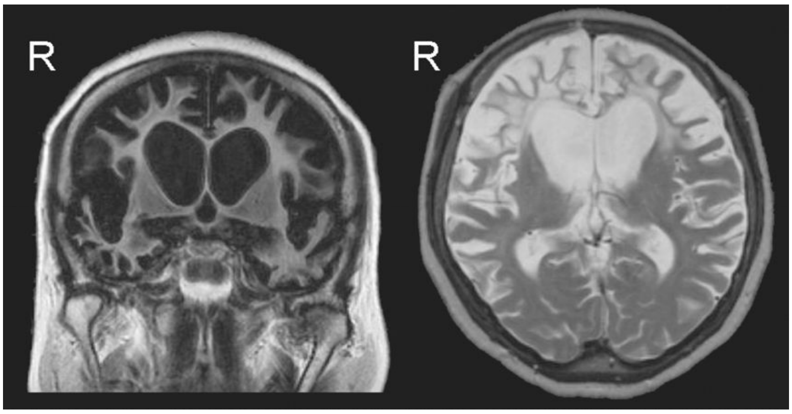

Fig. 1. Magnetic resonance imaging reveals dense atrophy in the frontotemporal lobe; however, the parieto-occipital lobe remains well-preserved. The left image is of the FLAIR MRI images of coronal section of the brain; the right image is a T2-weighted horizontal section.

her Mini-Mental State Examination (MMSE) [6] score was 1/30 (only the "repetition" test was correct) and her Mental Function Impairment Scale (MENFIS) [9] score (72/78) showed severe deterioration of several functions, with the exception of topographical disorientation. At that time, neurological examination showed a strong grasping reflex in both the hands of the patient (the left hand more so than the right) and compulsive laughter. Magnetic resonance imaging (MRI) revealed dense atrophy of the bilateral frontotemporal lobe, but the parieto-occipital lobe was well-preserved (Fig. 1). Single photon emission computed tomography (SPECT) imaging also showed bilateral frontotemporal hypoperfusion (the right side more so than the left). Frontal atrophy was severe, and she progressed to a complete loss of spontaneous activity; nevertheless, some residual functions were observed. For example, she was able to navigate certain areas in her neighbourhood; not only could she take a walk without losing her way, she could also take a different route every time. She particularly preferred the narrow back streets rather than the main street. She also could take a meal independently using a pair of chopsticks skilfully, but over and over again she picked up residual tiny food particles on the bowl. She even demonstrated good skills with a pair of scissors and unusual geometrical capabilities. We examined the last point in detail in the following section.

## 3. Methods and results

### 3.1. Cutting a line-drawing

When the examiner placed a pair of scissors and a piece of paper with a simple line-drawing (e.g. a star shape) in front of the patient, she grasped them and immediately started cutting the paper without any particular instruction. Her cutting behaviour was reasonable and accurate; she started at the end of the ideal extension line originating from an actual line (Fig. 2A) and cut out the figure, leaving a narrow blank space (usually within a millimetre). When the examiner placed a complicated figure (e.g. an overlap of several figures, such as a circle, rectangle, and triangle), she cut the figure regardless of form.

These results imply that although in daily living situations she showed a severe loss of voluntary activity and grasping reflex, her spontaneous activity and hand-motor skills were still well preserved.

### 3.2. Cutting a blank paper I

When the examiner placed a pair of scissors and a blank A4-sized piece of paper in front of the patient, she began cutting the paper without any instruction. In this situation, not only did she cut the paper at a right angle, but also repeatedly cut the paper for twelve trials. A thorough investigation revealed that the width of each paper cut was different for every trial (Fig. 1B). In addition, not including the first trial, the ratio of remaining paper to cut paper was consistently 0.2 until the seventh trial (Fig. 1C).

### 3.3. Cutting a blank paper II

At a later date (August 2007), when the examiner presented her with a blank A4-sized piece of paper and a pair of scissors, she took them and cut the paper straight into a rectangle, then repeatedly sliced the

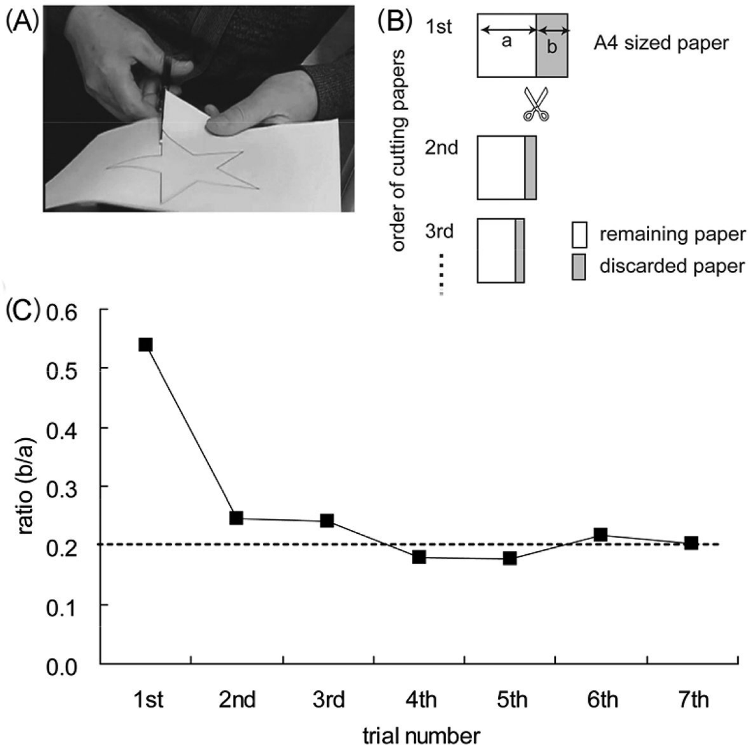

Fig. 2. A) The patient's cutting behavior is very reasonable and accurate. B) The patient cut the paper, using a pair of scissors, with a fixed ratio for each successive trial. The grey sections (b) represent the discarded paper and the white section (a) represents the paper remaining after cutting. C) The successive order of the ratio (b/a) showed that, with the exception of the 1st trial, the ratio was fixed at approximately 0.2.

length of the rectangle. Superposing the top and bottom of the rectangle revealed that the widths of the rectangle were almost identical. When the widths of each side of the rectangle were measured using an electric slide caliper (minimum measurable width is 0.01 mm, error of measurement is 0.01 mm), the difference was less than 0.01 mm.

## 4. Discussion

*Frontotemporal lobar degeneration* (FTLD) is the term applied to patients who present with primary dementia characterized by disproportionate atrophy of the anterior frontal and temporal lobes [8]. Previous reports have shown that some patients with FTLD presented several latent or newly developed abilities after onset of the disease. Most cases involved visual art, such as painting [5,7,14,15,18,21], but composing music and solving a puzzle were also reported [16,17]. In such reported cases, crucial lesions were attributed to the unilateral left-sided frontotemporal lobes. To date, there are no reports concerning geometrical abilities associated with dementia; therefore, we think that our patient's characteristic cutting behaviour might be a novel symptom. In addition, the bilateral lesions have never been reported to affect residual function, and thus we also think that this is the first reported case of a residual function of bilateral frontal lobe atrophy.

In this report, we described a case of FTLD and presented the following three characteristics: First, in the MR images, severe atrophy was observed in the bilateral frontal lobes and anterior portion of the temporal lobes, whereas parietal and occipital lobes were well-preserved, as well as the posterior portion of the temporal lobes. The patient's clinical symptoms, such as the declining spontaneous activity and grasping reflexes, corresponded with the MRI results. Second, although severe frontal lobe atrophy robbed her of many spontaneous activities, she nevertheless displayed good hand-motor skills. When given a pair of scissors and shown a figure drawn on paper, she spontaneously took the scissors and carefully cut around the drawing. Third, when she cut a plain piece of paper, she showed two geometrical preferences: equal ratio and parallel shape. In addition, the patient's parallel shape preference was unusually accurate.

Previous reports have shown that patients with a frontal lobes lesion shows several spontaneous activities when they are presented to objects without any instructions, such as putting glasses on patients' nose or pouring water from the bottle into the glass. These spontaneous activities have been called "utilization behaviour" [12,13]. The origin of utilization behaviour have been postulated as absence of a supervisory system in the frontal lobes and automatic activation of an action schema in the parietal lobes [20]. Therefore, in the present case, the sight of a pair of scissors and paper might activate her residual actions associated with the objects, such as taking a pair of scissors, taking a paper, and cutting the paper with the scissors. In a similar way, we have considered that a large part of her ADL might be caused by utilization behaviour. For example, she took a meal using chopsticks skilfully. This is because the sight of dishes and a pair of chopsticks would activate her skilful eating behaviour. However, her eating behaviour was not normal but deviated. Even when she finished everything on her dish, over and over again, she picked up tiny food particles on the bowl with dexterity. It is believed that the onset of her performance was based on utilization behaviour but, in addition to this, her performance was modified by additional abilities, such as hypersensitivity for local information. We believed that this might be true for her cutting behaviour.

The relationship between the posterior portion and geometrical ability has been suggested during studies of the line orientation test. For example, Benton et al. [3] found that the frequency of impaired performance on the line orientation test is particularly high in patients with right posterior parietal lesions. The line orientation test is a widely used neuropsychological test to assess visuospatial processing [11], in which the subject is asked to identify the orientation of pairs of lines on a multiple-choice display. Therefore, the posterior region appears to play a crucial role in recognizing the parallelism of diagonal lines of a rectangle.

Reports that musical or painting abilities are sustained or enhanced in some FTLD patients, have been explained by an emergence of residual right hemisphere functions released from the left hemisphere function due to atrophy [16–18]. The most plausible mechanism to explain sustained or enhanced abilities after onset of the dementia was hypothesized as a reflection of the paradoxical functional facilitation (PFF) effect [16,17]. The original notion of the PFF effect was proposed by Kapur [10] and, based on this hypothesis, Miller and his colleagues subsequently postulated that degeneration of the left temporal cortex led to decreased inhibition of "the right-sided and posteriorly located visual and musical systems" [16]. We also think that the PFF hypothesis could be extrapolated to fit our patient's symptoms; the emergence of the hidden posterior lobe function due to freedom from the frontal lobe occupation.

Concerning drawing abilities, it has been reported that some FTLD patients show excellent realistic drawing skills. Referring to the artistic abilities of autism patients [19], this kind of drawing ability has not been considered a result of a newly developed skill, but rather an expression of a hidden innate ability [4,15]. As such, we hypothesize that our patient's hypersensitivity for geometrical homogeneity might also be a result of expression of an innate, but newly expressed ability. In other words, her geometrical preferences imply that our brains, by nature, are affected by geometrical homogeneity.

Finally, when faced with severely demented patients, clinicians or caregivers might think that they cannot coax from them any sense of capability; however, our patient's case suggests even severe dementia patients might have several residual functions that could possibly be elicited.

## Acknowledgments

AM was supported by Chuo University Grant for Special Research and AM and MK was supported by Grant-in-Aid for Scientific Research from Ministry of Education, Culture, Sports, Science and Technology (MEXT). MK was also supported in part by a Showa University Grant-in-Aid for Innovative Collaborative Research Projects and a Special Research Grant-in-Aid for Development of Characteristic Education from MEXT.

## References

[1] L. Amaducci, E. Grassi and F. Boller, Maurice Ravel and right-hemisphere musical creativity: influence of disease on his last musical works? *Eur J Neurol* **9** (2002), 75–82.

[2] American Psychiatric Association: Diagnostic and statistical manual of mental disorders. 4th ed. Washington, DC: American Psychiatric Association 1994.

[3] A.L. Benton, N.R. Varney and K. Hamsher, Visuospatial judgement: A clinical test, *Arch Neurol* **35** (1978), 364–367.

[4] A. Chatterjee, The neuropsychology of visual artistic production, *Neuropsychologia* **42** (2004), 1568–1583.

[5] V. Drago, P.S. Foster, D. Trifiletti, D.B. Fitzgerald, B.M. Kluger, G.P. Crucian et al., What's inside the art? The influence of frontotemporal dementia in art production, *Neurology* **67** (2006), 1285–1287.

[6] M.F. Folstein, S.E. Folstein and P.R. Mchugh, "Mini-mental state". A practical method for grading the cognitive state of patients for the clinician, *J Psychiatr Res* **12** (1975), 189–198.

[7] S. Franklin, P. Van Sommers and D. Howard, Drawing without meaning? Dissociations in the graphic performance of an agnosic artist, in: *Mental lives: Case studies in cognition*, R. Campbell, ed., Oxford, UK: Blackwell, 1987.

[8] T.J. Grabowski and A.R. Damasio, Definition, clinical features and neuroanatomical basis of dementia, in: *The Neuropathology of Dementia*, (2nd ed.), M.M. Esiri, V.M.-Y. Lee and J.Q. Trojanowski, eds, Cambridge: Cambridge University Press, 2004, pp. 1–33.

[9] A. Homma, R. Niina, T. Ishii and K. Hasegawa, Development of a new rating scale for dementia in the elderly: Mental Function Impairment Scale (MENFIS) (in Japanese), *Jpn J Geriatr Psychiatry* **2** (1991), 1217–1222.

[10] N. Kapur, Paradoxical functional facilitation in brain-behaviour research. A critical review, *Brain* **119** (1996), 1775–1790.

[11] M.D. Lezak, Neuropsychological assessment. 3rd ed. New York: Oxford University Press 1995.

[12] F. Lhermitte, 'Utilization behaviour' and its relation to lesions of the frontal lobes, *Brain* **106** (1983), 237–255.

[13] F. Lhermitte, B. Pillon and M. Serdaru, Human autonomy and the frontal lobes. Part I: Imitation and utilization behavior: a neuropsychological study of 75 patients, *Ann Neurol* **19** (1986), 326–334.

[14] J.C. Mell, S.M. Howard and B.L. Miller, Art and the brain: the influence of frontotemporal dementia on an accomplished artist, *Neurology* **60** (2003), 1707–1710.

[15] A. Midorikawa, T. Fukutake and M. Kawamura, Dementia and Painting in Patients from Different Cultural Backgrounds, *Eur Neurol* **60** (2008), 224–229.

[16] B.L. Miller, K. Boone, J.L. Cummings, S.L. Read and F. Mishkin, Functional correlates of musical and visual ability in frontotemporal dementia, *Br J Psychiatry* **176** (2000), 458–463.

[17] B.L. Miller, J. Cummings, F. Mishkin, K. Boone, F. Prince, M. Ponton et al., Emergence of artistic talent in frontotemporal dementia, *Neurology* **51** (1998), 978–982.

[18] B.L. Miller, M. Ponton, D.F. Benson, J.L. Cummings and I. Mena, Enhanced artistic creativity with temporal lobe degeneration, *Lancet* **348** (1996), 1744–1745.

[19] L. Selfe, Nadia. A case of extraordinary drawing ability in an autistic child. London: Academic Press 1977.

[20] T. Shallice, P.W. Burgess, F. Schon and D.M. Baxter, The origins of utilization behaviour, *Brain* **112** (1989), 1587–1598.

[21] H. Tanabe, Y. Nakagawa, M. Ikeda, M. Hashimoto, N. Yamada, H. Kazui et al., Selective loss of semantic memory for words, in: *Brain Processes and Memory*, K. Ishikawa, J.L. McGaugh and H. Sakata, eds, Amsterdam: Elsevier, 1996, pp. 141–152.
